# Premenstrual syndrome: consultation sources and the impact on women's quality of life

**DOI:** 10.4314/ahs.v22i1.10

**Published:** 2022-03

**Authors:** Ruba M Jaber, Asma O Alghzawi, Hadeel H Salameh

**Affiliations:** 1 Assistant professor Family medicine/ Department of Family and community medicine, University of Jordan, Amman, Jordan. Family Medicine consultant, Women and Child's health specialist; 2 MD, Family Medicine Resident, University of Jordan Hospital, University of Jordan

**Keywords:** Premenstrual syndrome, quality of life, consultation

## Abstract

**Introduction:**

The main aim of this study was to explore the sources of consultation that women seek during premenstural syndrome (PMS), and to establish the association between the severity of PMS and how it affects the quality of life.

**Methods:**

Cross sectional study of 179 female patients, using an Arabic validated version of the shortened premenstrual assessment form, and a 7-item quality of life questionnaire that was developed by the researchers. The questionnaire was tested for validity and reliability via a pilot study before the initiation of data collection. Data was analyzed using SPSS version 19.

**Results:**

179 women participated in the study, with more than half of them aged between 20–30 years old. PMS prevalence was 88%; patient's predominantly sought help from their relatives (51%), followed by physicians (34%). There was no association found between the severity of premenstrual symptoms and seeking consultation. PMS symptoms affected women's daily activities (p-value 0.039), their satisfaction with their general appearance (p-value 0.001) and weight (p-value 0.022), and affected their relationships with family members (p-value 0.001) and other people (p-value 0.002).

**Conclusion:**

PMS is a common condition that affects women and their quality of life in several ways. Physicians and primary health care providers must be more vigilant in detecting the presence of PMS.

## Introduction

Premenstrual syndrome (PMS) is a common health problem in women of reproductive age, The American College of Obstetrics and Gynecology defines PMS as a complex set of physical and emotional symptoms that occur repeatedly in a cyclical manner one to two weeks before menstruation, and then disappear after the menstrual cycle^1^. One recent meta-analysis of PMS demonstrated the pooled prevalence of PMS to be 48%, ranging from 10% in a study conducted in Switzerland to >98% in one Iranian study^2^. This high-pooled percentage should emphasize the importance of studying the impact of such a common, chronic problem on women's quality of life (QoL) and productivity.

PMS symptomatology includes both physical and emotional symptoms; somatic symptoms associated with PMS include feeling overwhelmed, food cravings, insomnia or hypersomnia, headaches, pelvic pain and discomfort, breast tenderness and swelling, joint pain and bloating. In addition, irritability, anxiety, depression, mood swings, hostility, poor concentration, confusion, social withdrawal and interpersonal conflicts are the most commonly described psychological symptoms^3^–^5^. Many hypotheses have addressed the cause of PMS, including the effect of progesterone on opioids, catecholamine, serotonin, increased prolactin or increased sensitivity to prolactin, alteration in glucose metabolism and various other complex and multifactorial causes^6^. Regardless of the exact cause of PMS, the importance of its symptomatology and the impact of such recurrent symptoms on an affected woman's life cannot be negated. In fact, it has been estimated that affected women experience almost 3,000 days of severe symptoms during their reproductive years^7^.

PMS symptomatology may affect not only the individual but also their family and society as a whole^6^; several studies have described the increased incidence of child disturbance and family violence in families where the mother suffers from severe PMS^8^–^10^. Another previous study has provide substantial evidence that premenstrual symptoms have an effect on the daily life activities of women with PMS^11^.

Severe PMS symptoms typically interfere with the functional capacity of women, thus affecting multiple settings, such as home, school and work^1^. A significant association was found between mean premenstrual severity and functional impairment in a previous study. Schmelzer et al, stated that the severity of premenstrual affective syndrome was related to social impairment and the severity of psychological symptoms was correlated with occupational impairment^5^. Premenstrual complaints have proven to cause decreased labor productivity and quality of occupational life, economical losses and an increase in accident potential^12^. Indeed, women in their reproductive years have an important role in society, and they have tremendous responsibilities in terms of their families and communities, thus their QoL and work ability is of utmost importance to society as a whole. Notably, improvements in health-related QoL lessen the consequences associated with PMS, or at least they are perceived as more tolerable^12^.

In a recent study conducted by Hamaideh et al. among students and workers in one Jordanian university, the prevalence was found to be 80.2%^1^. This high prevalence indicates the importance of elucidating the impact of PMS on the QoL of these women. The aim of the current study was firstly to explore the sources of help that women seek in response to PMS, and secondly to elucidate the association between the severity of PMS and the QoL of affected women.

## Subjects and Methods

This cross-sectional study was conducted at a family medicine walk-in clinic in a hospital in Amman, the capital of Jordan. Patients at this hospital are primarily medically insured through the Ministry of Health, universities, and many affiliated public institutions and companies, although there are also some private patients. The clinics serve patients of all age groups who attend the clinic complaining of acute, sub-acute, and chronic complaints, as well as those who attend for health checkups and preventive services.

A convenience sample of 179 women was recruited. Women attending the clinic for various reasons between February 2018 and November 2018 were asked to complete a self-administered questionnaire. Inclusion criteria were all women aged 15–45 years who attended the clinic in the aforementioned period and who agreed to participate in the study. Women were excluded from the study if they were pregnant, had given birth in the last 6 months, or were on hormonal contraception, as each of these factors may affect the occurrence and severity of PMS^13^. A trained research assistant was responsible for obtaining consent from patients, explaining the purpose of the study, distributing the questionnaire, ensuring that all questions were answered, and helping fill out the questionnaires for patients with literacy problems or poor eyesight. Ethical approval was obtained from the appropriate ethical committees.

The questionnaire consisted of three main parts. The first asked about sociodemographic variables, including age, education level, average income, marital status and occupation. The second part of the questionnaire consisted of a modified shortened premenstrual assessment form (SPAF). This form, developed by Allen, McBride, and Pirie^14^, was used to determine the degree of premenstrual symptoms. The 10-item SPAF was used to assess the presence and/or changes in intensity of symptoms that are typically expressed during the luteal phase of the menstrual cycle. The SPAF provides the same assessment as the original 95-item Premenstrual Assessment Form, as demonstrated by its equally strong reliability (test-retest coefficient range of 0.6–0.7) and validity (internal consistency coefficient of 0.95)^15^. Owing to linguistics reasons, during translation for the current study, we divided the first item in the questionnaire into two questions; accordingly, patients were asked about breast pain and tenderness in one question and breast enlargement in another question. We also added two more items as per our literature review, including the appearance of acne on the face, upper chest, or back, and headaches^13^, ^16^, ^17^. As the SPAF is scaled from 0 to 3 according to the severity of the symptoms (0, no symptoms; 1, mild symptoms; 2, moderate symptoms; and 3, severe symptoms), a diagnosis of PMS was based on a PMS score of >14, with the symptoms preceding menses. PMS scores of 0–14 were rated as ‘no PMS, 15–29 as mild PMS, 30–44 as moderate PMS, and >45 as severe PMS. Patients were subsequently asked which consultation sources they have sought for such symptoms if present.

The third part of the questionnaire was developed by the researchers and involved questions about seven items to examine the effect of the presence of PMS symptoms of the women's QoL. The seven items included: performance of daily activates, missed work or school days, satisfaction with their general appearance, satisfaction with their weight, satisfaction with their eating habits, and their relationships with family members and with others. The final Arabic version of our modified SPAF, and the supplementary 7 QoL questions, were examined by three full professors and three assistant professors in the appropriate medical departments. All suggested changes were discussed and resolved, then the final Arabic version of our modified SPAF and 7 QoL questions were examined for reliability in a pilot study of 40 patients who were not included in the present analysis. The analysis revealed the questionnaire to be reliable (Cronbach's α of 0.747).

Data were analyzed using SPSS (version 19). The χ2 test was used to compare the frequencies of categorical variables; an independent t-test, analysis of variance, and linear correlation were used to compare continuous data as appropriate. P-values were considered significant at p<0.05.

## Results

In the current study, we found that 88% of our study population suffered from some degree of PMS; 50% had mild PMS, and only 2% suffered from severe PMS symptoms. Regarding the socio-demographics (shown in table 1) of our sample, >50% of the study population were aged 20 to 30 years old. Of the women included, 50% were single, >65% held Bachelor degree, and ∼40% worked full time.

**Table 1:** Socio-demographic characteristics of the sample

Sociodemographic variable	Frequency	Percent
Age Groups		
15–20	20	11.2%
21–25	55	30.9%
26–30	37	20.8%
31–35	33	18.5%
36–40	20	11.2%
41–45	13	7.3%
Social status		
Single	91	50.8%
Married	80	44.7%
Widowed	1	0.6%
Divorced	6	3.4%
Other	1	0.6%
Educational level		
Illiterate	2	1.1%
Elementary	5	2.8%
High School	38	21.2%
Bachelor's Degree	117	65.4%
Higher education	17	9.5%
Income/month		
Less than 800	103	58.2%
800–1500	57	32.2%
More than 1500	17	9.6%
Occupation		
Student	51	28.7%
Housewife	51	28.7%
Full time job	71	39.9%
Part time job	2	1.1%
Other	3	1.7%

**Figure 1:**
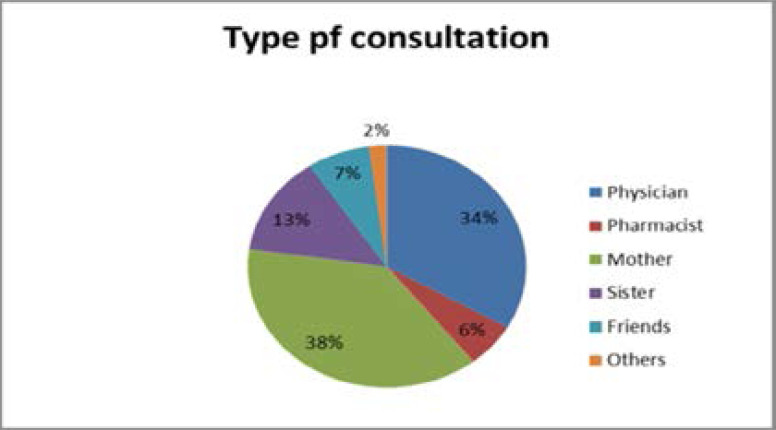
sources of consultations regarding PMS symptomatology

Figure (1) demonstrates the sources of consultation that women with PMS may seek; we found that >1/3 of the study sample (38%) sought help from their mothers, followed by 61 patients (34%) who sought advice from physicians, 23 (13%) asked their sisters, 12 (7%) consulted their friends, while 11 (6%) asked a pharmacist for advice, and 4 patients (2%) responded that they would consult the internet.

As outlined in table 2; no statistical significant difference was found between the severity of PMS symptomatology and seeking advice for those symptoms.

**Table 2:** The table represents the relation between severity of PMS and seeking consultation

PMS * Consult						Sig
			Did you consult anyone about your symptoms		Total	
			No	Yes		
PMS	No PMS	Count	39	39	78	0.194
		%	52.0%	37.9%	43.8%	
	Mild	Count	30	50	80	
		%	40.0%	48.5%	44.9%	
	Moderate	Count	6	12	18	
		%	8.0%	11.7%	10.1%	
	Severe PMS	Count	0	2	2	
		%	.0%	1.9%	1.1%	
Total		Count	76	103	179	
		%	100.0%	100.0%	100.0%	

As illustrated in table 3, which describes the effect of PMS symptoms on women's QoL, the mean score of the effect of PMS symptoms on the performance of daily activities was 3.3/4 (∼83%), which was statistically significant (p-value of 0.039). Women with severe PMS scored 3.5/4 in terms of the effect of PMS symptoms on their satisfaction about their general appearance and weight (p-values of <0.001 and 0.022 respectively).

**Table 3:** Represent the relation between the severity of PMS symptoms and the effect on dailyQoL items

Quality of life item		Number of patients	Mean PMS score	Sig
Perform Daily activities	No PMS	78	2.40	0.039
	Mild	80	2.81	
	Moderate	18	3.33	
	Severe PMS	2	3.00	
	Total	178	2.69	
Missed work or school	No PMS	78	1.62	0.113
	Mild	80	1.60	
	Moderate	18	1.94	
	Severe PMS	2	2.50	
	Total	178	1.65	
Satisfied with general appearance	No PMS	78	1.71	0.000
	Mild	80	2.13	
	Moderate	18	3.00	
	Severe PMS	2	3.50	
	Total	178	2.04	
Satisfied with your weight	No PMS	78	2.21	0.022
	Mild	80	2.55	
	Moderate	18	3.50	
	Severe PMS	2	3.00	
	Total	178	2.50	
Satisfied with your eating habits	No PMS	78	2.14	0.200
	Mild	80	2.25	
	Moderate	18	2.78	
	Severe PMS	2	3.50	
	Total	178	2.27	
Relationships with other	No PMS	78	1.63	0.002
	Mild	80	2.00	
	Moderate	18	2.39	
	Severe PMS	2	3.50	
	Total	178	1.89	
Relationship with family members	No PMS	78	1.76	0.001
	Mild	80	2.28	
	Moderate	18	2.72	
	Severe PMS	2	3.50	

In addition, when studying relationships with family members and with other people, we found that both were adversely affected by the presence of severe PMS symptoms; in both cases the mean score of the effect of the symptoms on the aforementioned items was 3.5/4 (87.5%), which was statistically significant (p-values of 0.002 and 0.001 respectively).

On other hand, PMS symptoms do not seem to affect women in terms of days absent from work or school, nor their satisfaction with their eating habits.

## Discussion

In the current study, >50 of the sample reported that they asked their mothers and sisters for help regarding PMS symptoms, which may be attributed to cultural issues; where family is still regarded as the first source of consultation in Jordanian families, particularly for menstrual cycle-related concerns related, which are considered embarrassing for females. Sisters sharing the same home environment malead to females depending on their families' experiences of PMS symptomatology. Physicians and pharmacists were found to be the source of consultation in >1/3 of our study group, which may reflect the severity of their symptoms, in that women feel they need to be evaluated by a healthcare worker. Our current findings are consistent with some published data from different parts of the world. One recent study conducted by Anandha et al. on female medical students on India, found that only 9.7% of the study sample would consult a physician or a pharmacist regarding their symptoms18. On the other hand, Tollossa et al6 reported that 48% of their sample, in a study that was conducted in Ethiopia, have sought medical treatment for their symptoms; in a Malaysian study, 19% of the study sample took prescribed treatment from their health professionals^19^.

The findings of this study demonstrated a significant correlation between moderate to severe PMS and limitations in the performance of daily activities, such as shopping, cooking and doing daily domestic activities. These results are consistent with the findings of Johnson 1987 and, Robinson and Swindle 200020, 21 as they confirmed the functional impairment effect of PMS symptomatology. A consistent relationship between premenstrual symptoms and social and occupational impairment was proven in their work; a similar result was also demonstrated in a study conducted in United Arab Emirates by Rizk et al in 2006, which indicated that PMS has a suggestive influence on QoL22. Comparable results were concluded in a study conducted in Korea, which stated that 14.3% of women reported that PMS symptomology had a significant impact on daily life activities23. Similar results were also reported in other studies conducted in India and Turkey18, 24. These results may be explained by the fact that daily activities can be postponed to a period when PMS symptoms have resolved.

PMS symptoms were not demonstrated to be associated with missing work or school days in this study, which is not consistent with the findings of a recent Jordanian study conducted by Hamaideh et al. This previous study stated that women with severe premenstrual symptoms reported higher rates of absence from work than those who reported less severe symptoms. Another study conducted in India, which investigated female medical students, reported that ∼16% of the students had symptoms that were severe enough for them to be absent from college18. This discrepancy between our results and those of the aforementioned studies may be attributed to the sample studied. Our study included women from different backgrounds and work environments, whereas the aforementioned studies were both conducted on college students and workers. In our culture, as it is in many developing countries, symptoms without physical evidence, such as fever or menorrhagia, are not considered enough for doctors to give sick leave certificates to patients, which may also explain why women do not miss days from their school or work for such problems.

We found that women with moderate to severe PMS were markedly unsatisfied with their general appearance and weight, which may be attributed to fluid retention that affects body weight and appearance. There is scanty literature about the effect of PMS on women's satisfaction with their weight and general appearance, even though it is one of the most frequently encountered symptoms. Our findings may highlight the need for more investigation into these important QoL factors.

Women with severe PMS symptoms reported that PMS symptoms affected their relationships with family members and other people; these results were expected, particularly when considering that the most common emotional symptoms of premenstrual syndrome include depression, irritability, anxiety, tension, crying, oversensitivity, feeling out of control, and mood swings1. Social withdrawal and decreased activity levels are also behavioral symptoms of PMS25, 26. One of the most common psycho-behavioral symptoms experienced by the participants of a study conducted on female university students in Ethiopia was loss of interest in doing things 6, these feelings, along with the somatic symptoms of PMS (such as abdominal cramps, fatigue, abdominal bloating, acne, and weight gain) exert a substantial impact on women's ability to deal with different people throughout their daily life.

## Conclusion

PMS is not only a chronic, recurrent problem that affect women in their reproductive years, it also has a severe impact on women's productivity and daily living activities. The current study highlighted the importance of this and the low rate of medical consultations sought for such a disruptive issue. These findings should urge healthcare providers to suspect and explore more about the presence of PMS symptoms and to take the initiative to diagnose and offer help for patients at need. Among the general population, urging the initiation of healthcare campaigns to increase the awareness and treatability of PMS would be beneficial.
